# Comparative genomics: From genotype to disease phenotype in the leishmaniases

**DOI:** 10.1016/j.ijpara.2007.05.015

**Published:** 2007-09

**Authors:** Deborah F. Smith, Christopher S. Peacock, Angela K. Cruz

**Affiliations:** a Immunology and Infection Unit, Department of Biology/Hull York Medical School, University of York, Heslington, York YO10 5YW, UK; b Pathogen Sequencing Unit, The Wellcome Trust Sanger Institute, Hinxton, Cambridge CB10 1SA, UK; c Departamento de Biologia Celular e Molecular e Bioagentes Patogênicos, Faculdade de Medicina de Ribeirão Preto, Universidade de São Paulo, Brazil

**Keywords:** *Leishmania*, Comparative genomics, Disease phenotype, Retrotransposons, RNAi

## Abstract

Recent progress in sequencing the genomes of several *Leishmania* species, causative agents of cutaneous, mucocutaneous and visceral leishmaniasis, is revealing unusual features of potential relevance to parasite virulence and pathogenesis in the host. While the genomes of *Leishmania major*, *Leishmania braziliensis* and *Leishmania infantum* are highly similar in content and organisation, species-specific genes and mechanisms distinguish one from another. In particular, the presence of retrotransposons and the components of a putative RNA interference machinery in *L. braziliensis* suggest the potential for both greater diversity and more tractable experimentation in this *Leishmania Viannia* species.

## 1 Introduction

Species of the genus *Leishmania* cause a diverse spectrum of infectious diseases, the leishmaniases, in tropical and subtropical regions of the world (reviewed in Murray et al., 2005). Belonging to the order Kinetoplastida and the family Trypanosomatidae, mammalian-infective species of the genus can be divided into two subgenera, *Leishmania (Leishmania)* and *Leishmania (Viannia)*, which differ principally in the site of parasite development within the female sandfly vector (reviewed in Sacks and Kamhawi, 2001). Once flagellated metacyclic promastigote stages are transmitted from vector to mammalian host, parasites enter resident tissue macrophages and transform into aflagellated replicative amastigotes, often inducing immuno-inflammatory responses and persistent tissue infection. The fate of these intracellular parasites determines disease type, which can range from cutaneous or mucocutaneous infection to diffuse cutaneous or visceral disease. The principal factors contributing to disease outcome are the infecting parasite species and immunological status of the host, including a well-established role for host genetic variation (reviewed in Lipoldova and Demant, 2006). Attributing the relative importance of these factors to disease outcome in humans is a complex area, however, particularly given the overlap in pathology associated with infection with different *Leishmania* species (Guerbouj et al., 2001;McMahon-Pratt and Alexander, 2004; Wilson et al., 2005; BenSaid et al., 2006). While multiple species cause cutaneous disease (see Table 1) and only three are usually considered as visceralising types (*Leishmania donovani*, *Leishmania infantum* and *Leishmania chagasi*), cutaneous lesions attributed to these latter species have also been reported (Gramiccia et al., 1995; Noyes et al., 1997; Gumy et al., 2004; BenSaid et al., 2006), while isolated cases of visceral disease due to cutaneous species have been recorded as well (Barral et al., 1991). Variable disease phenotype has also been observed in naturally and experimentally infected animals. Teasing out the relative importance of all contributing factors to clinical disease is a challenging objective.

A number of parasite factors affecting pathogenesis in the host have already been well-characterised. These include the surface zinc metalloprotease GP63 (or leishmanolysin) which protects promastigotes against host lysis by cleavage of complement, enhances phagocytosis and has recently been shown to protect against antimicrobial peptide-induced apoptotic killing (reviewed in Yao et al., 2005; Kulkarni et al., 2006); several parasite-derived cysteine proteases which can function as virulence factors (Mottram et al., 2004) and the complex glycoconjugates, lipophosphoglycan and the proteophosphoglycans, which promote parasite survival in the vector and host by a range of mechanisms (reviewed by Beverley and Turco, 1998; Ilg, 2000; Sacks and Kamhawi, 2001; Turco et al., 2001; Descoteaux and Turco, 2002; Lodge and Descoteaux, 2005).

Sequencing the genomes of representative parasite species provides a global framework within which to investigate the contribution of these and other parasite factors to the diverse forms of leishmaniasis and address the question: what is the contribution of parasite genotype to disease phenotype?

## 2 Sequencing the *Leishmania* genomes: which species?

The decision to initiate sequencing programmes for three kinetoplastid organisms, *Leishmania*, *Trypanosoma brucei* and *Trypanosoma cruzi* (the Tritryp genome projects), was made in principal at a Oswaldo Cruz Foundation (FIOCRUZ) – World Health Organization Special Programme for Research and Training in Tropical Diseases (WHO/TDR) Parasite Genome Network Planning Meeting in Rio de Janeiro in 1994. Although the available technology for completion of such large genomes was lacking at that time, these projects were considered feasible given the organisation of a committed consortium of participating laboratories, sufficient funding and anticipated advances in sequencing technology. Thirteen years later, three completed *Leishmania* genomes (*Leishmania major*, *L. infantum*, *Leishmania braziliensis*) are in the public domain (Ivens et al., 2005; Peacock et al., 2007), with sequencing of a fourth (*Leishmania mexicana*) in progress (http://www.genedb.org/). These first four species were chosen to represent the main species complexes of the *L. Leishmania* subgenus together with the best-characterised species of the *L. Viannia* subgenus. The strains selected were all infective, allowing study of host responses in suitable rodent models in vivo, and adapted for maintenance and manipulation in the laboratory (Laurentino et al., 2004; Ivens et al., 2005; Denise et al., 2006). Importantly, the three completed genomes represent species that usually give rise to distinct disease types (Table 1).

*Leishmania major* was the first species to be sequenced and has provided the model for subsequent genomic analyses. *L. major* is a causative agent of cutaneous leishmaniasis (CL), the most common disease form that is usually self-healing in humans but leaves unsightly scars in those affected (Table 1). Widespread in Africa and Asia, CL caused by *L. major* is predominantly a zoonotic infection, with parasites maintained in rodent reservoir hosts following transmission by phlebotomine sandflies. In the laboratory, *L. major* has been one of the principal experimental species used to dissect the roles of T_H_1/T_H_2 cells in response to infection in susceptible and resistant hosts (reviewed in Sacks and Noben-Trauth, 2002).

The case for sequencing a visceralising species of *Leishmania* as the second genome to be analysed was overwhelming: visceral leishmaniasis (VL) is the most serious disease form and frequently fatal if left untreated. Closely related species of the *L. donovani* complex are found in different geographical regions: *L. donovani* is the primary cause of VL in the Indian subcontinent and East Africa, *L. infantum* in the Mediterranean region and *L. chagasi* in the New World (Table 1). Humans are the only known reservoir of *L. donovani* while canines, especially domestic and stray dogs, provide the reservoir hosts for *L. infantum* and *L. chagasi*. While the last two species are generally considered to be genetically identical (Mauricio et al., 2000), all three species are very similar as demonstrated in recent comprehensive phylogenetic studies using both non-coding and protein-coding sequences (Kuhls et al., 2005, 2007; Mauricio et al., 2006; Lukes et al., 2007). The choice of a prevalent *L. infantum* strain for genome sequencing was made on the basis of its virulence in animals, transmissibility in sandflies and adaptability to laboratory experimentation (Denise et al., 2006).

The New World species *L. braziliensis*, within the subgenus *L. (Viannia)*, is the third and most divergent species sequenced to date – and the principal cause of CL and mucocutaneous leishmaniasis (MCL) in tropical America. Originally associated with close contact with the forest environment, it is now accepted that *L. braziliensis* transmission may occur in peri-domestic and domestic habitats. More recently, studies on the reservoir hosts strongly suggest that a number of rodent species are involved, including domestic rats (Grimaldi and Tesh, 1993; Brandao-Filho et al., 1994; Oliveira et al., 2005).

The most severe pathology associated with this species is the disease affecting mucous membranes, mainly the anterior nasal region and occasionally the pharynx and larynx, which can result in destruction of the cartilagenous tissue. Most frequently, mucosal disease occurs after the appearance of cutaneous lesions and its diagnosis may happen months or years after treatment of the primary lesion (Zajtchuk et al., 1989). However, recent clinical investigation indicates that mucosal infection may be present, but not diagnosed, concomitantly with the primary cutaneous lesion (Boaventura et al., 2006). This is a relevant issue because the patient usually does not recognise MCL until partial destruction of the nose mucosa and/or cartilage occurs. Treatment of such cases is often difficult and death may occur as a result of uncontrollable secondary infections.

## 3 The *L. major* genome

The *L. major* genome is composed of both nuclear and independently replicating mitochondrial or kinetoplast DNA. The structure and function of kinetoplastid DNA have been reviewed recently (Lukes et al., 2005) and will not be discussed further here.

**Fig. 1:**
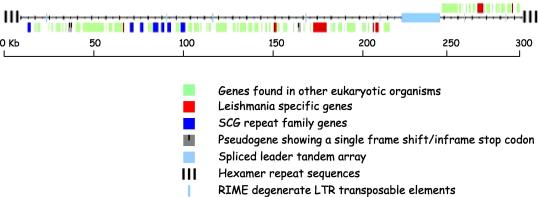
The structure of a typical *Leishmania* chromosome: chromosome 2 of *Leishmania major*. The location and coding strands of the 74 protein-coding genes are shown as coloured boxes, coded according to the categories indicated below. The majority are genes conserved with other eukaryotes; distribution of the *Leishmania*-specific and scβ-galactosyltransferase (SCG) repeat family genes are also shown, together with three pseudogenes. Other chromosomal features include the telomeric hexamer repeats, the spliced leader tandem array and the ribosomal mobile element (RIME) degenerate LTR transposons.

Prior to publication of the Tritryp kinetoplastid genomes in 2005, relatively little was known about the overall physical architecture of kinetoplastid chromosomes. However, earlier studies on antigenic variation in *T. brucei* had defined telomeric expression sites for variant surface glycoprotein (VSG) as polycistronic transcription units (reviewed in Pays et al., 1994), while work on RNA processing mechanisms in *T. brucei* and *Leishmania* had demonstrated linkage between 3′-polyadenylation sites and 5′-*trans*-splicing of adjacent genes, suggesting a coupled mechanism that could be processive along a chromosome (LeBowitz et al., 1993; Ullu et al., 1993; Matthews et al., 1994). The first whole chromosome sequence of *L. major* (Myler et al., 1999) revealed an unusual pattern of gene distribution, consistent with the polycistronic transcription model. Of the 79 identified genes, 29 were present as a contiguous unit on one DNA strand with the remaining 50 as a second unit on the opposite strand. Further chromosome sequencing revealed that the genes on other *L. major* chromosomes are similarly aligned in long arrays that appear to be transcribed as a single unit (Martinez-Calvillo et al., 2003; see Fig. 1) prior to *trans*-splicing and polyadenylation. These directional gene clusters (DGCs) range in size from a few to hundreds of genes stretching over 1 Mb of DNA. DGCs are separated by AT-rich strand switch regions (SSRs) believed to contain sites for transcription initiation and termination (Martinez-Calvillo et al., 2004). Whole genome microarray experiments have recently confirmed that most genes are constitutively transcribed in *Leishmania* (Holzer et al., 2006; Leifso et al., 2007). Differential gene expression between different life cycle stages is subsequently mediated by downstream processing events affecting mRNA stability and translation (Boucher et al., 2002; Myung et al., 2002).

The DGC type of chromosomal organization is remarkably well-conserved in all the kinetoplastids sequenced to date, with most of the *L. major* chromosomes being completely syntenic with corresponding regions in the larger *T. brucei* chromosomes (El-Sayed et al., 2005a), despite separation of these two species by at least 200 million years of evolution (Stevens et al., 2001). DGCs do not contain clusters of genes of related function similar to the organisation of prokaryotic operons, however.

In contrast to the high conservation of DGC gene order shared by *L. major* and the other Tritryps, organization of the *L. major* chromosome ends is distinctive, with short and highly conserved subtelomeric regions bordering distinctive repeat sequences that precede heterogeneous telomeres (Ivens et al., 2005). Interestingly, there are no large subtelomeric multigene families encoding proteins involved in host immune evasion – these are features not only of the *T. brucei* and *T. cruzi* genomes but also of other protozoan pathogen genomes such as those of *Plasmodium* spp. (Hall et al., 2002). Associated with this lack of telomeric repeated genes is a lack of transposable elements in the *L. major* genome (Bringaud et al., 2006) – these mobile sequence units are associated with gene re-arrangements in the subtelomeric regions of *Trypanosoma* spp. (Berriman et al., 2005).

## 4 The *L. major* genome – gene content

The 33 Mb genome of *L. major* is predicted to contain ∼8,300 genes, a figure likely to be accurate given the distinct codon bias in the coding regions and the rarity of *cis*-splicing in this species, features that make gene prediction reliable (Ivens et al., 2005; Peacock et al., 2007). Within this gene complement are a number of tandemly arrayed gene families, a kinetoplastid feature that may overcome the limitations of polycistronic transcription by increasing gene dosage (see below). Properties of the *L. major* genome, in comparison with the *L. infantum*, *L. braziliensis* and *T. brucei* genomes, are listed in Table 2.

The *L. major* genes can be divided into three broad categories. Around 85% encode the core proteome common to all kinetoplastids. Many of these function in metabolism and can be described as house-keeping genes, similar to those found in higher eukaryotes. A second category contains genes restricted to kinetoplastids, with no orthologues in higher eukaryotes. These include sequences encoding cytoskeletal proteins, RNA editing components and other metabolic enzymes. A third category appears to be restricted to *Leishmania*: these number ∼1,000, ∼12% of the genome (El-Sayed et al., 2005a). Many of these genus-specific genes have no conserved or recognisable functional domains and it is reasonable to predict that some of them function in host/pathogen interactions.

Annotation of the *L. major* genome benefited considerably from the comparative genomics approach developed in the Tritryp projects: based on chromosomal position and sequence similarity, orthologues of genes experimentally characterised in the trypanosomes could be identified in *Leishmania* and vice versa. This approach was used to great effect in the identification of *L. major* metabolic pathways, which are more extensive than in either *T. brucei* or *T. cruzi* (Berriman et al., 2005). *Leishmania* metabolism reflects parasite lifestyle in the vector and the host and is influenced by the availability of nutrients in both locations (reviewed in Zilberstein, 1993; Tielens and Van Hellemond, 1998). As with the other Tritryps, *Leishmania* lacks the biosynthetic pathways for essential amino acids which must therefore be host-derived, explaining the presence of the large amino acid transporter gene families found in *L. major* (Ivens et al., 2005). Similarly, while all the Tritryps encode the full machinery for uptake and degradation of glucose (Berriman et al., 2005), *L. major* alone appears to have the capability to hydrolyse disaccharide sugars, probably as a consequence of sandfly nectar feeding that provides an alternative source of energy for the promastigote stage of the parasite (Muller and Schlein, 2004). *L. major* also encodes enzymes required for haem synthesis, which are lacking from the other Tritryps (Ivens et al., 2005). Haem is an important *Leishmania* growth factor that is required in both extracellular and intracellular stages of the life cycle (Chang and Chang, 1985). The last three enzymes in the biosynthetic pathway, encoded solely by *Leishmania*, share a high similarity to genes in prokaryotic pathways, like many of the apparent kinetoplastid gene acquisitions that lead to new metabolic activities – in the Tritryp analysis, as many as 50 genes were identified as likely candidates for lateral gene transfer from prokaryotic sources (Berriman et al., 2005). It should be noted, however, that synthesis of haem has not been demonstrated biochemically in *Leishmania* and genes coding for enzymes required for the rest of the pathway have yet to be identified.

While *L. major* lacks the highly expanded and divergent large subtelomeric immune evasion gene families common to other protozoan pathogens, the sub-telomeric regions are not totally devoid of genes, with some sequence families found in these locations (see Fig. 1). The largest and best studied gene family codes for a family of phosphoglycan scβ-galactosyltransferases (SCGs). These enzymes are involved in side chain modifications of the phosphoglycan repeats of the major promastigote glycoconjugate, LPG (Dobson et al., 2003). There are 14 members spread across three subfamilies (*SCG*, *SCGR* and *SCGL*) but seven of the “core” *SCG* genes are found exclusively at distinct subtelomeric sites (Dobson et al., 2006). Interestingly, SCG expression does not appear to be restricted to a single location that might support an antigenic variation model in *Leishmania*, similar to that operating with the *T. brucei* VSGs (reviewed in Barry and McCulloch, 2001). Further analysis is required to rigorously test this “single-cell exclusive-expression” model (Dobson et al., 2006). It is clear, however, that these sub-telomeric locations can facilitate gene conversion events that may ultimately support the strain-specific diversity in LPG galactosylation known to be critical for successful sandfly transmission of different *Leishmania* strains and species (reviewed in Sacks and Kamhawi, 2001; Kamhawi, 2006).

In contrast to the sub-telomeric *SCG* genes, the tandemly arrayed gene families coding for *Leishmania* surface proteins are found at internal chromosome sites. A good example is the *GP63* gene family which encodes glycoproteins critical for host invasion and virulence (Joshi et al., 2002; Yao et al., 2003). These zinc metalloproteases are not confined to *Leishmania* species however: a single tandem *GP63* gene array is present in all three Tritryp species (El-Sayed et al., 2005a), while recent studies suggest that GP63-type proteins are conserved and functional in insect and plant trypanosomatid species (Masini Masini d’Avila-Levy et al., 2006) as well as the more distant *Trichomonas vaginalis* (Carlton et al., 2007).

In *Leishmania*, the *GP63* locus is limited to four to six copies in *L. major* and *L. infantum* but expanded approximately eightfold in the *L. braziliensis* genome (Peacock et al., 2007). Studies with multiple isolates of *L. braziliensis* and *Leishmania peruviana* (both *L. Viannia* spp., see Table 1) have shown extensive size variation and evidence of gene re-arrangement at this locus during long-term maintenance in the laboratory, suggesting that the *GP63* locus is susceptible to dynamic change in this sub-genus (Victoir et al., 1995). This does not seem to be the case in a more limited study in *L. major* (Sadlova et al., 2006). Recent analysis of sequence diversity within a single *GP63* gene copy in multiple *L. donovani* complex strains has confirmed earlier speculation that evolution of this family is influenced by mosaic gene conversion and provides strong genetic evidence for GP63 as an important marker for host preference in infection (Mauricio et al., 2006).

Another family of leucine-rich membrane surface proteins, the PSA-2 or GP46 family, is restricted to *Leishmania* spp.; analysis at both the DNA and protein levels failed to detect GP46 in the *L. braziliensis* complex (McMahon-Pratt et al., 1992). Confirming this earlier study, only one *PSA-2*-like gene has been annotated in the *L. braziliensis* genome. This is in contrast to the genome strains of *L. major* (with 25 *PSA-2* genes) and *L. infantum* (also with a large gene complement, although the precise copy number has been difficult to determine accurately). The PSA-2s are known to bind to host cell macrophages and protect the parasite from complement-mediated lysis during infection with *L. chagasi* and *L. infantum* (Kedzierski et al., 2004; Lincoln et al., 2004). In *L. infantum*, different strains that cause either visceral or cutaneous disease show considerable correlative variation in the size of the repeat regions containing the *PSA-2* genes (Guerbouj et al., 2001).

The amastin genes, the largest family in the *Leishmania* genome, encode small surface-expressed glycoproteins of as yet unknown function that are conserved in *T. cruzi*. Importantly, a majority of these genes are expressed specifically in intracellular amastigotes in *L. major* and therefore presumably contribute to survival in the host (Rochette et al., 2005). Unlike most other large *Leishmania* gene families, the amastin genes are not present in a single array but found on seven different chromosomes in the *L. major* genome (Ivens et al., 2005). Most of these sites contain small groups of genes (one to four copies) but two large arrays are also present, with the largest containing amastin genes alternating with the highly conserved tuzin genes; a similar arrangement is found in *T. cruzi* but is absent from *T. brucei* (El-Sayed et al., 2005b). The second large gene array, not present in *T. cruzi*, contains amastin genes separated by degenerate tuzin genes. A likely explanation for this gene complexity is that a common kinetoplastid ancestor possessed a single amastin/tuzin array, which underwent duplication, re-arrangement and diversification after separation of the *Leishmania* and *Trypanosoma* lineages. With the exception of a lone copy on *L. major* chromosome 36, *L. infantum* and *L. braziliensis* have amastin gene arrays at the same locations, although sequence read depth suggests that the large amastin array with intact tuzin genes is shorter in *L. braziliensis*.

Other *Leishmania*-specific proteins that are not necessarily surface-exposed may also be important regulators of host–parasite interactions. One interesting example is an apparent homologue of the human cytokine macrophage inhibitory factor (MIF). The first cytokine to be identified, MIF is also one of the most pleiotropic, implicated in a wide range of cellular activities that could influence parasite survival (Calandra and Roger, 2003). Addition of purified recombinant human MIF to *L. major*-infected macrophages activates parasite killing by inducible nitric oxide synthase and TNF-α (Juttner et al., 1998). In malaria, variations in the expression of MIF have been associated with susceptibility to high density parasitemia, while reduced MIF production may lead to increased severity of disease (Awandare et al., 2006). MIF-deficient mice also show increased susceptibility to *L. major* (Satoskar et al., 2001), highlighting the important role of this cytokine in mounting an effective immune response to infection.

MIF sequences are not confined to the host, however. All three *Leishmania* spp. sequenced to date have two similar *MIF* gene copies located at a strand switch region (see http://www.genedb.org/). Functional analysis of these sequences may be of considerable interest, given that MIF expression in the filarial worm *Brugia malayi* influences the progression of the CD4+ T cell response to infection towards a T_H_2 pathway (Falcone et al., 2001). The *Leishmania MIF* genes show greatest similarity to bacterial orthologues, suggesting that they too may have been acquired by lateral gene transfer following diversification from the trypanosome lineage.

## 5 Comparison of the *L. major*, *L. infantum* and *L. braziliensis* genomes

From an evolutionary perspective, phylogenetic analyses have suggested a neotropical origin for the *Leishmania* genus (Stevens et al., 2001) and, while there has been some controversy in this designation (see Kerr, 2000), this has been largely resolved in a recent multifactorial genetic study (Lukes et al., 2007). Irrespective of this debate, *L. braziliensis* is the most genetically and biologically divergent of the three sequenced species. Divergence between the *Leishmania* species complexes is estimated to have occurred 15–50 million years ago (Lukes et al., 2007), within the same range as two potential host species, mouse and human. Given this period of isolation, it was expected that there would be significant differences in both genome architecture and gene repertoire between *L. braziliensis*, *L. infantum* and *L. major*. Indeed, while the genomes have a similar DNA content of around 33 Mb, karyotypic differences had already been identified by linkage group analysis (Britto et al., 1998): *L. major* and *L. infantum*, in common with other Old World species, have a haploid content of 36 chromosomes, while the New World species have either 35 (*L. braziliensis* complex) or 34 (*L. mexicana* complex). These differences were shown to be due to fusion of pairs of chromosomes (chromosomes 20 + 34 in *L. braziliensis*; chromosomes 8 + 29 and 20 + 36 in *L. mexicana*), with the former observation now confirmed in the sequencing project.

**Fig. 2:**
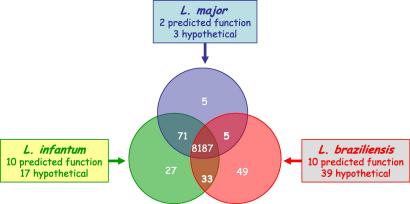
*Leishmania* species-specific genes. The Venn diagram shows how many of the 8,187 protein-coding genes are species-specific or shared between two of the three sequenced *Leishmania* species. These genes are subdivided into those that have a predicted function (mostly through sequence identity) and those that are of unknown function at the time of publication (the “hypotheticals”).

Surprisingly, comparison of the respective orthologous chromosomes has revealed remarkable conservation of both gene content and gene order in all three genome species. Despite the differences in gene copy number within some of the major protein-coding families described above, not a single chromosomal re-arrangement has been identified between *L. major* and *L. infantum* across the whole genome, while *L. braziliensis* has only a few possible sequence re-arrangements (Peacock et al., 2007). Equally surprising, from the total content of ∼8,300 genes in each species, only ∼200 can be identified as differentially distributed between the three genomes. The most divergent, *L. braziliensis*, possesses 47 genes that are absent from the other two species. In comparison, *L. infantum* has 27 species-specific genes while *L. major* has only five (see Fig. 2). A number of the other differentially distributed genes are found in two out of the three species. Some of these species-specific sequences have already been analysed at the molecular level. Examples include the *L. infantum A2* gene that encodes an amastigote-specific repeat-containing protein previously characterised in *L. donovani*, the only *Leishmania* sequence to date that confers a change in virulence phenotype when introduced into *L. major* by genetic transfection (Zhang et al., 2003); and the *HASP* and *SHERP* genes, expressed from a single locus (absent in *L. braziliensis*) in infective stages of *L. major* and *L. infantum*, with their protein products localizing to the plasma membrane and intracellular membranes, respectively, in these species (Denny et al., 2000; Knuepfer et al., 2001).

In the Tritryp genome analyses, most genes specific to each of the representative species were found either at the ends of the DGCs or in the subtelomeric regions of the chromosomes, regions that appear to be more tolerant to genome re-arrangement. However, comparison of the three *Leishmania* genomes has revealed that gene variation is not predominantly restricted to the subtelomeric regions or even the SSRs but is evenly distributed across the genome (Peacock et al., 2007).

*Leishmania* is also distinctive from other eukaryotes in the apparent mechanism by which species-specific gene variation occurs. Whereas insertions/deletions and sequence re-arrangements play major roles in gene diversification in most other eukaryotes characterised to date, degeneration of existing genes (leading to probable loss of function) accounts for ∼80% of the species differences in *Leishmania*. These degenerate sequences have in-frame stop codons and frame shifts, generating truncated open reading frames that are presumably not translated. One example is the gene encoding cysteine peptidase *Pfp1*, which is present as an intact gene and translated in *L. major* (Eschenlauer et al., 2006). However, there are five in-frame stop codons and a frame shift in the *L. infantum* orthologue, while the syntenic region in *L. braziliensis* is even more degenerate.

*Pfp1* like some of the other species-specific genes, appears to be another candidate for lateral gene transfer from bacteria. Of the remaining species-specific sequences not caused by loss of function, many also fall into this category. One example is the cyclopropane fatty acyl synthase (*CFAS*) gene, present in *L. infantum* and *L. braziliensis* but absent from *L. major*. Acquisition of novel genes in this way may be a mechanism for environmental adaptation to promote survival; similar adaptations to stress or other stimuli may lead to the redundancy of other sequences clearly identified as pseudogenes in the *Leishmania* genomes (Peacock et al., 2007). In the case of *CFAS*, acquisition of this gene may have an impact on parasite survival in the host, since the CFAS orthologue in *Mycobacterium tuberculosis* is associated with increased virulence and persistence, functions that apparently require cyclopropanation of a mycolic acid substrate in the bacterial cell wall (Rao et al., 2005).

Despite its chromosomal plasticity (Martinez-Calvillo et al., 2005), the incredible conservation of synteny revealed by comparative genomic analyses of these three species suggests that the *Leishmania* genome is highly stable and has not undergone major genomic re-arrangements during speciation. One contributing factor to this stability could be a lack of mobile DNA elements, as originally demonstrated in the *L. major* genome and verified in *L. infantum*. The comparative sequencing project has revealed some surprising observations, however, one of the most striking being the presence of transposable elements in *L. braziliensis*.

## 6 Retrotransposons in *L. braziliensis*

Repeat sequences are a common feature of both prokaryotic and eukaryotic genomes, with different categories contributing 50% or more to some eukaryotic genomes. Among these groups are the mobile genetic elements which, by virtue of their ability to move to different chromosomal locations, are considered important in shaping the course of genome evolution. Mobile elements are widespread in the biological kingdom. When actively proliferating (Wickstead et al., 2003), they can create and destroy or modify existing genes and regulatory elements, thus reshaping the structure and function of their host genomes. When no longer able to proliferate (“dead repeats”), they are still important markers for evolutionary events, a palaeontological record.

Although some protozoan parasites carry no mobile elements or any vestige of them (Katinka et al., 2001; Carlton et al., 2002; Gardner et al., 2002; Xu et al., 2004), different types of transposable elements have been found in kinetoplastids. The genomes of *T. brucei* and *T.cruzi* contain both highly reiterated interspersed elements (Bhattacharya et al., 2002; Wickstead et al., 2003; El-Sayed et al., 2005b) and retroelements, transposons that require reverse transcription from an RNA intermediate. Depending on their mechanism of transposition, these retroelements are classified either as long terminal repeat (LTR) retroelements (or retrotransposons) or non-LTR retroelements (the retroposons), which include the short and long interspersed nuclear elements (Singer, 1982).

In some kinetoplastids, retrotransposons (VIPER), site-specific retroposons (SLACS, CZAR, CRE) and non-site-specific retroposons (Ingi and L1Tc) have been described (Kimmel et al., 1987; Murphy et al., 1987; Martin et al., 1995; Teng et al., 1995; Wickstead et al., 2003). *Trypanosoma cruzi* bears the highest number of repetitive sequences, which occupy at least 50% of the parasite genome. Long terminal repeat (LTR) and non-LTR retroelements account for ∼5% and 2% of the haploid *T. cruzi* and *T. brucei* genomes, respectively (El-Sayed et al., 2005b). These mobile elements probably played an important role in shaping these parasites’ genome organization and evolution.

In contrast, analysis of several *Leishmania* species originally indicated a lack of active retroelements (Bhattacharya et al., 2002). In the *L. major* and *L. infantum* genomes, it was possible to detect remnants of retroposons, named DIREs (degenerate ingi/L1Tc-related elements), which had been previously described in trypanosomes (Ghedin et al., 2004; Bringaud et al., 2006). Other relics of retroelements, distinct from *ingi*/L1Tc, were also detected in *L. major*. These are conserved in some Old and New World *Leishmania* species, but absent from the *T. cruzi* and *T. brucei* genomes (Pedrosa et al., 2006).

Against this background, in addition to these degenerate retroelements, it was surprising to detect potentially active retroposons in the genome of *L. braziliensis* (Peacock et al., 2007). Similar to the organization described for the SLACS and CZAR elements in *T. brucei* and *T. cruzi*, these non-LTR retrotransposons are associated with the spliced leader array in *L. braziliensis* (Aksoy et al., 1987; Villanueva et al., 1991).

In addition, the organization of the *L. braziliensis* telomeres is remarkably distinct from the Old World species. They accommodate the so called TATEs (Telomeric Associated Transposable Elements), a family of 20–30 novel DNA transposable elements, each including putative reverse transcriptase, phage integrase (site-specific recombinase) and DNA/RNA polymerase domains. These are highly conserved retroelements with a precise site of insertion within the telomeric hexamer repeats and a typical TT duplication flanking the transposon. The clusters of tandemly arrayed TATEs at the ends of chromosomes resemble ribosomal mobile element (RIME)/Ingi retroelements that are also organised in clusters within the subtelomeric regions of *T. brucei* chromosomes (Bringaud et al., 2002; Peacock et al., 2007). Based on current annotation of the *L. braziliensis* genome, TATEs are present on at least 12 chromosomes, precisely inserted in the middle of the GGGTTA repeats (Peacock et al., 2007). The TATE-containing *L. braziliensis* chromosome ends share longer stretches of sequence with a higher number of heterologous chromosomes, compared with the corresponding chromosomes in *L. major* (Brito, L.O. et al., unpublished data). On the other hand, only remnants of TATE elements are detected at *L. braziliensis* internal chromosomal sites. Besides the bias of abundance of these elements at the chromosome ends, selection against disruption of coding regions at chromosome central domains could explain TATE distribution patterns. To understand the role of the TATEs in shaping the *L. braziliensis* genome, it will be necessary to improve genome assembly and further analyse TATE distribution in different strains and species of the *Viannia* subgenus.

## 7 RNAi in *L. braziliensis*

Among different functional classes of non-coding RNAs, there is a large class of post-transcriptional regulators implicated in gene expression control in various organisms. Two classes of small RNAs, the short interfering RNAs (siRNAs) and the microRNAs (miRNAs) have been extensively studied in gene silencing. Initially described in *Caenorhabditis elegans* (Lee et al., 1993; Wightman et al., 1993), the term RNA interference (RNAi) was used to describe these events (Fire et al., 1998) and link them to similar post-transcriptional silencing phenomena in plants and fungi (Jorgensen, 1990; Romano and Macino, 1992).

Currently, RNAi is the best-known mechanism of gene silencing, an evolutionary conserved process in which double-stranded RNA induces the sequence-specific degradation of homologous mRNA (Fire et al., 1998). Besides its role in the control of endogenous mRNAs, the process seems to have an important defensive role against viruses and mobile element activity.

**Fig. 3:**
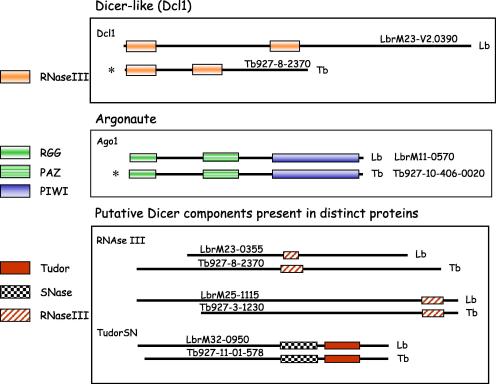
RNAi machinery: *Leishmania braziliensis* versus *Trypanosoma brucei*. Schematic representation of the main components of the RNAi pathways identified by sequence similarity and/or functional evaluation in *T. brucei* (Tb) and *L. braziliensis* (Lb). Comparisons of the gene products and their domains in each species are accompanied by their GeneDB gene IDs; Tb927, *T. brucei* genes; LbrM, *L. braziliensis* genes. Domain IDs are depicted on the left. RGG indicates a domain containing arginine-glycine-glycine repeats; SNase indicates the Staphylococcal nuclease domain present in TudorSN proteins. ^∗^Indicates genes confirmed to be functionally active in the *T. brucei* RNAi pathway.

An RNAi mechanism was rapidly identified in *T. brucei* (Ngo et al., 1998) but has not been demonstrated in other kinetoplastids including *L. major* and *T. cruzi* (Robinson and Beverley, 2003; El-Sayed et al., 2005b). Therefore, one of the most unexpected differences between the sequenced *Leishmania* genomes is the identification of genes implicated in the RNAi pathway in *L. braziliensis*. One of the hallmarks of this pathway is *Dicer* activity, which converts double-stranded RNA (dsRNA) into small interfering RNA (siRNA). Annotation of the genomes of three *Leishmania* species and the trypanosomes did not reveal a *Dicer* orthologue (Berriman et al., 2005; El-Sayed et al., 2005b; Ivens et al., 2005; Peacock et al., 2007). Instead, it was speculated that a combination of independent proteins carrying the relevant domains DSRBD, DEAD/H box RNA helicase and RNase III could be responsible for *Dicer* activity in *T. brucei*. Orthologues of genes carrying these domains have also been annotated in the *L. braziliensis* genome (see Fig. 3 for an outline of the putative RNAi machinery in *L. braziliensis*, compared with *T. brucei*).

Curiously, it has recently been shown that a *T. brucei Dicer*-like protein (TbDcl1), quite divergent from the typical *Dicer* proteins described for other eukaryotes, is required for the generation of siRNA-size molecules and for RNAi (Shi et al., 2006a). Interestingly, a TbDcl1 gene is also present in *L. braziliensis* (Peacock et al., 2007), although there are no orthologues in *T. cruzi*, *L. major* or *L. infantum*.

Another crucial component of the RNAi pathway is Argonaute, an endonuclease involved in the dsRNA-triggered cleavage of mRNA. Unlike *L. major* and *L. infantum*, *L. braziliensis* contains one orthologue for *Ago1*, the functional argonaute gene of *T. brucei*. Tb*AGO1* can be functionally replaced by the human gene *Argonaute 2*, which suggests that Tb*AGO1* has the endonuclease activity involved in mRNA target degradation in the trypanosome RNAi pathway (Shi et al., 2006b). The *L. braziliensis* gene contains the typical argonaute domains PAZ and PIWI, the latter containing conserved amino acid residues that are essential for functional TbAGO1 (Shi et al., 2004). In addition, an N-terminal RGG domain, present in TbAGO1 and shown to be essential for its association with polyribosomes, is also present in the *L. braziliensis Ago1* gene (Shi et al., 2004). Thus, the *L. braziliensis* gene product may also participate in the control of translation.

It is an intriguing observation that RNAi machinery is present in only one of the three, highly syntenic *Leishmania* species sequenced to date. While this could indicate a lack of biological relevance for this pathway in the parasite, a more interesting possibility is that the availability of RNAi in *L. braziliensis* plays a role in the biological differences observed between the two subgenera. The demonstration of RNAi and its biological consequences in *L. braziliensis* awaits further experimentation.

Whether RNAi machinery has been lost or acquired across the kinetoplastid species and genera is an open question. Examination of the syntenic regions in *L. major* and *L. infantum* identified remnants of *Argonaute* in both, an observation that favours the proposal that RNAi was lost in the subgenus *Leishmania*. This speculation is reinforced by the conservation of key RNAi members between organisms as distant as *T. brucei* and humans (Shi et al., 2006a,b). It is relevant to stress that RNA viruses have been detected in species of the subgenus *L. Viannia* (Guilbride et al., 1992; Widmer and Dooley, 1995), suggesting that *L. braziliensis* could have retained RNAi as an antiviral defense mechanism (although there is also a single report of an RNAi virus in *L. major*; Scheffter et al., 1995). In addition, RNAi could have a role in regulating the function of the *L. braziliensis*-specific transposable elements described above. A role for RNAi as an endogenous mechanism to hamper expression of specific genes and defend cells from viruses and mobile elements has been demonstrated in a range of organisms, including *T. brucei* (Wickstead et al., 2003; Ullu et al., 2004).

The discovery of a *Leishmania* RNAi mechanism could also be of practical importance, given the wide and effective use of inducible RNAi methods to study gene function in other eukaryotes. An RNAi system for reducing gene expression would be especially relevant in *Leishmania*, a diploid pathogen that lacks a sexual cycle, and provide an interesting alternative to the classical two-step gene knockout strategy (Cruz et al., 1991). RNAi has been rigorously tested for in *L. major* and *L. donovani* with no success (Robinson and Beverley, 2003). Detection of an RNAi machinery so similar to that of *T. brucei* in *L. braziliensis*, however, suggests that this mechanism may be shown to be a useful tool in *L. braziliensis*. A word of caution however: the genetic plasticity of the *Leishmania* genome, which impedes the generation of null mutants for essential genes, may also interfere with the outcome of RNAi experiments. In fact, downregulation of *T. brucei* essential genes by RNAi led to selection of cell lines that escaped from the deleterious effect of RNA interference (Ullu et al., 2004). Notwithstanding this concern, the combination of RNAi and classical reverse genetic approaches promises to bring novel insights into the functional biology of *Leishmania* parasites.

## 8 How do the genome sequences impact on our understanding of parasite tropism?

It is premature to speculate on how knowledge of the genome sequences of different *Leishmania* species will impact on our global understanding of disease phenotype. However the primary comparative analysis (Peacock et al., 2007) supports some preliminary observations. Firstly, the genes that differ between the three species are good candidates for functional analysis to determine their roles in establishment of infection. With the *A2* gene as a precedent (Zhang et al., 2003), it can be speculated that one or more of these sequences, expressed separately or together, may impact upon the course of disease. Testing this hypothesis will require the generation and functional testing of specific mutations in suitable models of infection. Murine models of CL are well-established, with experimental *L. major* infection reproducing distinct aspects of the clinical spectrum of human disease (reviewed in Gumy et al., 2004). The establishment of a dermal infection model, that introduces small numbers of infective *L. major* metacyclics into the host dermis via sandfly feeding, more closely mimics the natural course of parasite transmission (Belkaid et al., 1998). Murine models for *L. infantum* and *L. braziliensis* are not as well advanced, partly because these species are more difficult to grow and differentiate in vitro and partly because of the variable sensitivity of model mouse strains to infection with these parasites. Progress has recently been made, however, in the development of dermal models for both *L. infantum* and *L. braziliensis* infection (Ahmed et al., 2003; de Moura et al., 2005). For all species, more work is required to relate the immune correlates of protection in mice to those that are important in human and, as appropriate, canine infection.

Second, as most of the species-specific genes encode proteins sharing little identity with other eukaryotic sequences, the use of a combination of genetic, biochemical and immunological analyses will be essential to help delineate relevant biological functions. It will also be necessary to unravel the importance of those tandemly repeated genes that vary in copy number between species, given that this mechanism can support increased gene expression and potentially lead to detectable phenotypic effects. In this context, the recently published whole genome microarray analyses suggest that RNA expression is largely constitutive throughout the *Leishmania* life cycle with few genes showing increased expression at the RNA level in different parasite stages (Holzer et al., 2006; Leifso et al., 2007). Further studies are required to verify these observations in different parasite species and correlate RNA and protein expression levels, using high resolution proteomic methods (Foucher et al., 2006).

A third observation from the comparative genome analysis is that, despite the well-conserved synteny between the three model species, *L. braziliensis* may have the capacity for more extensive genomic reorganisation, given the putative retrotransposons and RNAi machinery found in this species. Clearly, whether RNAi actually operates as a functional mechanism in *L. braziliensis* awaits definitive experimental validation, while the functional relevance of the retroelements requires more extensive multiple strain analysis to determine possible levels of sequence variation. However, it cannot be discounted that retrotransposons play a role in shaping the *L. braziliensis* genome, impacting on the different disease outcomes associated with this species.

## 9 Future perspectives

Sequencing the genomes of three *Leishmania* species, with a fourth in progress, has provided a wealth of new information that is reshaping research initiatives for leishmaniasis. Extending this type of analysis to further species and virulent strains may reveal further surprises. Of most immediate practical application perhaps are the unlimited number of potential vaccine candidates that are now available and require robust evaluation, to identify the most favourable molecules to incorporate into clinical trials (Kedzierski et al., 2006). Similarly, the *Leishmania*-specific metabolic pathways revealed by comparative analysis are underpinning focused biochemical analyses to identify valid drug targets for development. New, more sensitive and specific diagnostic reagents for the leishmaniases should also emerge from exploring the different genome sequences.

More fundamentally, genomic knowledge is revealing fascinating insights into *Leishmania* biology. In particular, the current comparative analyses are generating testable hypotheses relevant to understanding the importance of parasite genotype in pathogenesis. It is possible that just a few species-specific parasite genes are crucial to disease outcome and/or alternatively, that expression levels of key conserved parasite genes differ considerably both between and within species (perhaps as a consequence of variation in gene copy number). It is also possible that the parasite genome may play only a minor role in determining disease phenotype. In-depth functional analyses may ultimately unravel the relative contribution of parasite genes to disease progression in this spectrum of crippling infections.

## Figures and Tables

**Table 1:** Human-infective species of the *Leishmania* genus

Old World species	New World species	Disease type
***L. major* complex**	***L. mexicana* complex**	Cutaneous
*L. (L.) major*	*L. (L.) mexicana*	
*L. (L.) tropica*	*L. (L.) amazonensis*	
*L. (L.) aethiopica*	*L. (L.) pifanoi*	
	*L. (L.) venezuelensis*	
	***L. (Viannia) subgenus***	
	*L. (V.) braziliensis*	
	*L. (V.) panamensis*	
	*L. (V.) guyanensis*	
	*L. (V.) peruviana*	
	*L. (V.) lansoni*	
		
	*L. (V.) braziliensis*	Mucocutaneous
		
*L. (L.) aethiopica*	*L. (L.) amazonensis*	Diffuse cutaneous
	*L. (L.) pifanoi*	
		
***L. donovani* complex**		Visceral
*L. (L.) donovani*		
*L. (L.) infantum*⁎	*L. (L.) chagasi*⁎	

The main species complexes and subgenus are shown in bold; the species with complete genome sequences either available or being generated are underlined.

⁎ Species that can also be associated with cutaneous leishmaniasis.

**Table 2:** Genome facts: the *Leishmanias* versus *Trypanosoma brucei*

	*Leishmania major*	*Leishmania infantum*	*Leishmania braziliensis*	*Trypanosoma brucei*
Total size (Mb)	32.8	32.1	32.0	26.1
Contigs	36	562	1,041	30
No. of chromosomes	36	36	35	11^∗^
Chromosome size range (Mb)	0.3–2.8	0.3–2.8	0.3–2.8	1–5.2
Overall G + C content %	59.7	59.3	60.4	46.4
No. of genes	8,298	8,154	8,153	9,068
No. of pseudogenes	97	41	161	904
Average gene size (bp)	1,894	1,868	1,873	1,592
Gene density (per Mb)	252	235	258	317
Coding percentage	48.0	44.0	48.5	50.5
Coding G + C content %	62.5	62.4	60.4	50.9
No. of DGCs	133	133	n/a	127
Average DGC length (kb/genes)	240/61	n/a	n/a	204/71
No. of tRNAs	83	62	66	65
No. of snoRNAs	693	n/a	n/a	353
No. of snRNAs	6	n/a	n/a	5
No. of rRNAs	63	n/a	n/a	56
Average intergenic size	1,939	2,049	1,976	1,279
Active mobile elements	None (degenerate RIME/DIRE)	None (degenerate RIME/DIRE)	TATEs, SLACS	ingi, RIME, DIRE, SLACs, SIRE, VIPER

Data included in this Table are correct as of February 2007 (for *Leishmania* species) and July 2005 (for *T. brucei*). *, only the megabase chromosome are included, not the intermediate or mini-chromosomes. DGC, directional gene cluster; RIME, ribosomal mobile element; DIRE, degenerate *ingi*/L1Tc-related element; TATE, telomere-associated transposable element; SLACS, Spliced Leader Associated Conserved Sequence; SIRE, short interspersed repetitive element; VIPER, LTR retroelement related to SIRE.
